# Pediatric advanced chronic kidney disease as a family challenge: coping and communication in daily life

**DOI:** 10.1007/s00431-026-06861-2

**Published:** 2026-03-25

**Authors:** Maria Agnes Jonas, Hendrik Napierala, Nele Kanzelmeyer, Christina Taylan, Nina Kubiak, Julia Thumfart

**Affiliations:** 1 Department of Pediatric Gastroenterology, Nephrology and Metabolic Diseases, Charité Universitätsmedizin Berlin, Corporate Member of Freie Universität Berlin and Humboldt Universität Zu Berlin, Berlin, Germany; 2 Department of Pediatric Respiratory Medicine, Immunology and Critical Care Medicine and Cystic Fibrosis Center, Charité Universitätsmedizin Berlin, Corporate Member of Freie Universität Berlin and Humboldt Universität Zu Berlin, Berlin, Germany; 3 Clinic for Internal Medicine, Psychosomatics/Psychotherapy, Charité–Universitätsmedizin Berlin, Corporate Member of Freie Universität Berlin and Humboldt Universität Zu Berlin, Berlin, Germany; 4 Institute of General Practice and Family Medicine, Charité Universitätsmedizin Berlin, Corporate Member of Freie Universität Berlin and Humboldt Universität Zu Berlin, Berlin, Germany; 5 Department of Pediatric Kidney, Liver, Metabolic and Neurological Diseases, Hannover Medical School, Hannover, Germany; 6 Department of Pediatric Nephrology, Faculty of Medicine, Children’s and Adolescents’ Hospital, University Hospital of Cologne, Cologne, Germany

**Keywords:** Chronic kidney disease, Family coping, Sibling experience, Psychosocial adaptation, Life limiting illness

## Abstract

Pediatric advanced chronic kidney disease (CKD) profoundly affects not only the patient but the entire family system. This study explores how affected families—including patients, siblings, and primary caregivers—perceive and experience advance CKD as a chronic, life-limiting illness with life-prolonging but non-curative treatment options. A qualitative study was conducted involving seven families recruited from three pediatric nephrology centers. Semi-structured interviews were conducted with patients with CKD ≥ 4, at least one sibling and one parent per family. Both, patients and siblings, were aged ≥ 14 years. Data were analyzed using qualitative content analysis. Five major themes were identified: families’ understanding of CKD as a chronic life-limiting disease, coping with CKD and its uncertain course, the perspectives of healthy siblings, family dynamics, and communication. Even in stable medical conditions like a functioning kidney transplant, participants acknowledged CKD as a lifelong condition without definitive cure options. Siblings frequently adapted flexibly to family needs, occasionally adapting their role to fill structural gaps. Family members involved in caregiving—whether parents or siblings—frequently expressed feeling insufficiently informed. In contrast, patients and siblings not engaged in caregiving generally felt well informed.

*Conclusion*: By capturing family-wide perspectives, this study contributes new evidence on intra-family dynamics and supports the need for family-centered care that integrates structured communication, sibling involvement in clinical practice, and screening for family-level risk factors.

**Table:** 

**What is Known:** • *Pediatric advanced CKD impacts family functioning.* • *Siblings are at risk for psychological distress but are often overlooked in care.*
**What is New:** • *Additional stressors beyond CKD can increase caregiving burden and compromise family coping.* • *Information needs are tied to the level of responsibility and involvement in disease management.* • *Siblings often adopt flexible roles to stabilize the family system.*

**Supplementary Information:**

The online version contains supplementary material available at 10.1007/s00431-026-06861-2.

## Introduction

Chronic kidney disease (CKD) in children, particularly in advanced stages (stage 4 or higher), is a life-limiting, irreversible condition with lifelong medical dependency and increased long-term mortality. Advanced CKD disrupts physical, emotional, and social development. Treatments such as dialysis or transplantation are life-prolonging but not curative, often limiting autonomy, peer interaction, and school life [1–4]. Studies show that children and adolescents with CKD have significantly lower health-related quality of life (HRQoL) than their healthy peers—and in some cases, even lower than those with other chronic conditions such as diabetes or congenital heart disease [5–7].

These burdens extend beyond the patient, affecting the entire family system. Parents frequently face overwhelming responsibilities and ongoing uncertainty, navigating complex treatment demands while managing work and family life. Siblings, in turn, must cope with disrupted routines and reduced parental attention [8–10].

Despite this systemic impact, studies focus on individual perspectives—typically those of the affected child, a single parent, or a sibling—rather than considering the family as a dynamic, interacting unit [8–10].

This qualitative study aims to address this gap by exploring how individual families—comprising patients, siblings, and parents—understand, experience, and cope with advanced CKD.

## Methods

### Study design

We employed a qualitative descriptive design based on individual semi-structured interviews and reported the study in accordance with the consolidated criteria for reporting qualitative research (COREQ) framework [11]. Ethical approval was granted by the ethics committees of Charité Universitätsmedizin Berlin (EA2/234/17).

### Setting and participants

Families were recruited through three German pediatric nephrology centers in collaboration with parent associations. We applied a multi-step purposive sampling strategy: Eligible participants were selected through post-transplant outpatient clinics, dialysis units, treating clinicians, and parent associations.

Inclusion criteria required that (1) one child had CKD stage ≥ 4; (2) the family included at least one sibling and one parent willing to participate; (3) both the affected child and the sibling had to be ≥ 14 years. A requirement mandated by the ethics committees and chosen to ensure that participants possessed adequate cognitive and emotional maturity to engage in individual interviews on sensitive topics such as coping, family dynamics, and perspectives on life-limiting disease. Only German-speaking families were included.

No formal intellectual screening was conducted, and health practitioners referred only adolescents considered able to participate, including one patient with a mild developmental delay. A total of nine families were approached: seven participated.

Separate, one-to-one interviews were conducted with each participating family member. No other persons were present during any interview. Participants received written information about the study, including confidentiality assurances and their right to withdraw at any time. Written informed consent was obtained from all participants; for minors, parental consent was additionally required. Sociodemographic data were collected through a pre-interview questionnaire, including age, gender, CKD stage, time of CKD diagnosis, employment, household characteristics, and religious beliefs.

### Data collection

Data were collected between June 26, 2024, and October 29, 2024. Interviews were conducted by M.J., a medical student who underwent specific training and preparation for this study. None of the participants had prior relationships with the interviewer.

Interviews took place either in private rooms at the participating nephrology centers or by telephone, depending on feasibility. Whenever possible, all interviews of one family were conducted on the same day. At the start of each telephone interview, participants were asked to confirm that they were alone. Telephone interviews were chosen instead of video because of data protection regulations.

Interviews were conducted using a semi-structured interview guide developed by the research team based on insights from previous studies [12, 13] and established recommendations for qualitative research in the social sciences [14]. A multidisciplinary qualitative research team reviewed the guide to ensure alignment with the study’s objectives. The guide was tested in a pilot interview and subsequently revised; it was not included in the final analysis (see Supplemental Materials).

### Data analysis

All Interviews were audio-recorded, transcribed verbatim, anonymized for confidentiality and securely stored. Data were analyzed using Kuckartz’s structured qualitative content analysis, applying a combined deductive-inductive approach [15]. The deductive code frame was derived from the operationalization of our research question: Illness understanding, coping, family communication, and perspectives on life-limiting disease were translated into dimensions for the interview guide, which subsequently served as initial deductive categories. Inductive codes were added to capture themes emerging directly from the data.

Data analysis and coding were facilitated using MAXQDA© software (version 24). Initial coding of all transcripts was conducted by M.J. The subsequent analytic process involved continuous collaboration within the study team. A core research group of three members (M.J., a medical student, N.K., a psychologist, and J.T., a pediatric nephrologist) met regularly—every 2 weeks—to review, discuss, and refine the coding, ensuring consistency and analytic depth through iterative triangulation. In addition, a broader established qualitative multidisciplinary research group located at the Charité Universitätsmedizin, representing diverse professional backgrounds (including medicine, psychology, nursing, social work, and law) was consulted at selected points to provide external perspectives and strengthen interpretive rigor.

Coding was carried out at multiple levels: first at family level to preserve the integrity of each family as the primary unit of analysis; then by functional role (“patients,” “siblings,” and “parents”) to allow cross-role comparison; and finally, by topic, aligning the material with the thematic structure that emerged during analysis.

We evaluated sample adequacy using information power and thematic saturation. After analyzing the sixth family, no new themes emerged, one additional family was interviewed to confirm this. Recruitment was concluded once all eligible families had been approached. Member checking was not conducted due to the risk of introducing recall or confirmation bias.

## Results

### Participant characteristics

The final dataset consisted of interviews with seven families: seven patients, seven parents, and eight siblings, resulting in 22 individual interviews. Thirteen interviews took place face-to-face in private clinical rooms in the respective center; nine were conducted by telephone to accommodate participants’ preferences. Interview duration ranged from 15 to 106 min. Characteristics of all participants are provided in Table 1.

**Table 1:** Participant characteristics (*N* = 22)

	Parents (*n* = 7)	Patients (*n* = 7)	Siblings (*n* = 8)
Gender			
Female	5	4	6
Male	2	3	2
Age			
≥ 14–18	0	7	6
> 18–25	0	0	1
> 25–35	0	0	1
> 35	7	0	0
Employment			
Student	0	7	6
Full-time	5	0	2
Part-time	2	0	0
Underlying disease			
CAKUT	0	3	0
Metabolic disease (methylmalonic aciduria)	0	1	0
Atypical hemolytic uremic syndrome	0	1	0
Unknown etiology	0	2	0
CKD stage			
Stage ≥ 4, waitlisted and in dialysis	0	2	0
Post-TX	0	5	0
Time of diagnosis			
Since birth	0	3	0
Early age	0	3	0
Adolescence	0	1	0

### Themes

Five main categories emerged from the analysis regarding how families navigate CKD as a chronic condition: (1) comprehension of CKD as a chronic life-limiting chronic disease, (2) coping with CKD and its uncertain course, (3) social strain and family dynamics, (4) siblings’ perspectives, and (5) communication.

#### Comprehension of CKD as a chronic life-limiting disease (Table 2)

**Table 2:** Comprehension of CKD as a chronic life-limiting disease

Quote	Quotation	Participant
1a	“Well, (…) we know the kidneys are working now, but we also know they won’t work for a lifetime—meaning dialysis, another transplant, and everything that comes with it will come our way again.”	Parent 6
1b	“I accepted it (the disease), I’d say. From my point of view, it’s nothing terrible.”	Parent 4
1c	“(…) but now it (the illness) isn’t really a big topic anymore, because (Pat 5) is basically just a completely normal person—without illness, limitations, or disability.”	Sibling 5
1d	“Now that I have a kidney, I’m technically no longer really sick.”	Patient 1
1e	“And the worries are always there. She’s a sick child—she’s chronically ill. She’ll always be a child of concern. When (Pat 4) has a fever, all my alarm bells go on. We have to go to the hospital when she hits 38.5 °C. But when her siblings have a fever, I just say, okay, we’ll bring it down—it’ll pass. I take care of them too, but the level of stress is completely different.”	Parent 4
1f	“The illness really dominated our daily life. It still does, but at that time it was completely overwhelming. Everything revolved around it… Planning was impossible during that time; we were basically living in a constant state of uncertainty. The phone could ring at any moment”	Parent 5

Participant numbers correspond to family units (e.g., Patient 1, Sibling 1, and Parent 1 belong to the same family)

The majority of interviewed families had undergone a successful kidney transplantation and were interviewed in a prolonged stable phase of the disease. While all participants acknowledged that CKD is a lifelong condition without definitive cure options (quote 1a), their interpretation of this reality varied. Descriptions ranged from acceptance to denial—some family members equated post-transplant stability with the absence of illness and perceived the transplant as a temporary cure (quotes 1b–1d).

Most families maintained a constant awareness of CKD’s persistent impact. For them, the disease remained an ever-present concern, and the affected child was often perceived as the family’s “problem child.” Even minor health issues—such as a fever, which would typically not be concerning in a healthy child—were perceived as significantly more serious and distressing due to the underlying illness (quotes 1e and 1f).

#### Anticipating the fall: how families cope with CKD and its uncertain course (Table 3)

**Table 3:** Anticipating the fall: how families cope with CKD and its uncertain course

Quote	Quotation	Participant
2a	B: “Yeah, mostly because I’m a bit afraid that my kidney won’t last when I’m an adult—and that I’ll need a new one.” I: “What thoughts come up when you think about it?” B: “Going back on dialysis and waiting again.”	Patient 7
2b	“Yes. Fear, of course—it’s this uncertainty about how long things will keep going well, and that at some point there will be a moment when the kidneys just fail. Because it’s obviously not something that lasts forever. I’d say that’s one of the biggest fears.”	Sibling 6
2c	“I’m more the type of person who really starts dealing with things when the situation is actually there. (…) And I know that if it ever comes to him needing dialysis, then we’ll take things as they come and handle it.”	Parent 7
2d	“I don’t like thinking too much about things like that, because… It’s just that (Pat 4) is really close to me, and if I think about it too much, I just end up worrying all the time.”	Sibling 4.1
2e	“I generally don’t do that (thinking about the future), because I end up putting way too much pressure on myself. I prefer to just take my time and enjoy the time I have, instead of thinking about the future.”	Sibling 7
2f	“Well, (…) we know the kidneys are working now, but we also know they won’t last a lifetime—so that means dialysis, another transplant, and everything that comes with it will come back to us at some point. Living with that is hard. I know it’s there; like a sword hanging over you, without knowing when it’s going to fall. It’s like living with the handbrake on. I find it hard to fully let go.”	Parent 6

Participant numbers correspond to family units (e.g., Patient 1, Sibling 1, and Parent 1 belong to the same family)

The unpredictable course of CKD led to varied strategies to cope with the fear of progression among family members. A shared concern across all families was the fear of graft failure, re-dialysis, and the need for multiple transplantations (quotes 2a and 2b).

Some family members adopted a present-focused approach, choosing to concentrate on daily life and address medical challenges only when they arose instead of worrying about the future (quote 2c). Thinking about the disease and potential complications caused them so much psychological distress that they preferred to avoid the topic altogether and focus on enjoying the present (quotes 2 d and 2e).

Others were constantly worried about the future describing it being “like a sword hanging over you, without knowing when it’s going to fall. (…) it’s like living with the handbrake on somehow” (quote 2f).

Strategies in dealing with the disease course tended to be similar within each family (quotes 2c and 2e/2b and 2f).

#### Family roles and care responsibilities under social strain (Table 4)

**Table 4:** Family roles and care responsibilities under social strain

Quote	Quotation	Participant
3a	“And it was that moment again where I felt like I had to carry everything. I accompanied him to appointments and all that. I was the one expected to clean up the mess, and that wasn’t pleasant. It still isn’t. There’s still this expectation—if his transplant fails, then I’ll be the one to do it. And while I’ve already decided I would, the problem is that both of our parents just step back, assuming: “I will take care of it.” And it’s not even seen as something you should be grateful for—it’s just taken for granted. And that’s really frustrating.”	Sibling 1
3b	“Before, my sister was never really my focus. But after my parent passed away, it really felt like I took it upon myself to take on that responsibility.”	Sibling 2
3c	“My parents are problematic. They argue a lot. One of my parents has an issue with substance abuse during the period when I was dealing with my kidney problems, which was very stressful for me. That kind of thing always affects me deeply, no matter what. And my other parent is the kind of person who creates a lot of stress. Even when I was told that my blood pressure shouldn’t rise too much because it could be dangerous, she would often still cause stress. (…). I also had panic attacks in the hospital. That was really bad (…) The doctors say it’s because I’ve already been through so much with dialysis and everything. I always internalized the stress—that’s how it came out, yeah.”	Patient 1
3d	I really didn’t feel like dealing with the illness at first—but there was no way around it. So in a way, I *did* want to, because it was my child. That’s why I really immersed myself in all the layers and the complexity of what was going on. The social factors, the emotional factors, the psychological ones, and even the spiritual ones—everything that plays a role in life	Parent 5
3e	“Actually, I think it was even better that way—that I didn’t have too much contact with HCP—because it meant I didn’t felt completely involved, but rather like I was in a kind of safe observer position, so to speak.”	Sibling 6
3f	“I live pretty much like a normal person. I’m happy and content. The only thing that’s really annoying is having to sort the pills every week. But my family supports me very well. Honestly, I’m very satisfied with my life.”	Patient 7
3 g	“In terms of family—our close family has really been supportive. Especially the bond with my siblings is very strong, and they regularly visited (Pat 2). That helps a lot”	Sibling 2

Participant numbers correspond to family units (e.g., Patient 1, Sibling 1, and Parent 1 belong to the same family)

In two families, additional stress factors besides dealing with CKD could be identified. These seemed to affect the caregiving role of the parents and led to a redistribution of responsibilities within the family. In these cases, siblings assumed care-related responsibilities, while the affected children reported managing stress in isolation, experiencing panic attacks in connection with handling these situations without sufficient support (quotes 3a–3c).

In other families, where such challenges were not reported, parents retained the primary caregiving role (quote 3 d). Siblings reported satisfaction with their limited involvement in care and were able to pursue their own activities (quotes 3e). The affected children also reported fewer illness-related responsibilities and described aspects of everyday life as relatively normal (quote 3f). Across different family situations participants expressed that close emotional bonds within the family helped them manage stress (quote 3 g).

#### Siblings—“we are still here too” (Table 5)

**Table 5:** Siblings—“we are still here too”

Quote	Quotation	Participant
4a	“And yes, I’m often asked how (Pat 6) is doing, but no one asks about me. Sometimes I think, I can tell you how (Pat 6) is doing, but it would be nice if you also asked about me. Because when you’re talking to me, I’m the person you’re speaking to, not my sibling. So I would say this point — that we are still here too, that we are also important, and that we have our own problems and difficulties — is something I really want people to understand.”	Sibling 6
4b	“No one ever asked me about it (the sibling’s disease) — no one has ever really asked how I’m dealing with all of this.”	Sibling 4.2
4c	“(…) That was a really tough time for me. I missed being close to my parents, because they were often at the hospital with him.”	Sibling 5
4d	“Well, my brother is treated better than I am and I think it really depressed me that they were so worried about him.”	Sibling 1
4e	“When we were children playing outside with friends, especially with regard to (Pat 5), it was always said that we had to be careful so that no one kicked or punched his belly or caused any injury there— and that he would eventually be old enough to look after himself a bit, too. But overall, I never really… I don’t know… in the end everything always went well.”	Sibling 5
4f	But after my mother passed away, it was really the case that I took this responsibility upon myself	Sibling 2
4 g	“Especially since I was pushed into a role of responsibility within the family. There were situations where my mom didn’t have time to attend an appointment, so I stepped in. And then once again I had to be the responsible one who fills in—because she didn’t have time—but still wasn’t considered entitled to receive any information. That just feels wrong. My parents didn’t realize that this was a pressure situation for me”	Sibling 1
4 h	“I like it when she feels the same—she even tells me sometimes that she really sees me as her mom. That’s always a kind of reassurance for me.”	Sibling 2
4i	“I would say that throughout this whole time, I’ve developed a pattern of behavior. I tend to handle difficulties by myself rather than talking to someone about it, simply because I never really had those opportunities.”	Sibling 6
4j	“I grew up with it—for me, it was always the most normal thing in the world.”	Sibling 3
4 k	“It (being without the parents) didn’t feel that bad to me, because I actually thought it was cool to be at my grandparents’ place. I just didn’t really understand how serious it all was back then.”	Sibling 4.2
4 l	“There are really only siblings there and I always found that incredibly helpful. First, I learned that I’m not alone. You make connections there—but more than that, you learn a lot about yourself and about how to deal with certain situations. And you start to figure out how to help yourself.”	Sibling 6

Participant numbers correspond to family units (e.g., Patient 1, Sibling 1, and Parent 1 belong to the same family)

Siblings of children with CKD described feeling overlooked not only within the family system but also in their broader social environment (quotes 4a and 4b). Across all families, siblings of children with CKD reported receiving less parental attention due to the demands placed on the affected child—an experience that was described as emotionally difficult and, in some cases, even depressing (quotes 4c and 4 d).

A central pattern across the interviews was the way in which siblings adapted to family needs. We refer to this as a “*flexible role adaptation*” capturing how siblings shifted between different positions—ranging from taking on little responsibility—such as watching out during childhood play so the affected sibling would not get injured, to assuming substantial caregiving or even parental tasks when needed (quotes 4e–4 g).

Those more actively involved in caregiving described recognition from the affected sibling as something that gave them reassurance (quote 4 h).

During illness of the patient, some siblings develop independent behavior strategies and tend to solve problems on their own without confiding to someone (quote 4i). For some, growing up with a sibling with CKD was perceived as their normal reality (quote 4j). Alternative caregivers, such as grandparents, were perceived as something special and served as a resource to feel protected against the seriousness in the context of disease (quote 4 k).

Connecting with other siblings in similar situations helped to navigate through challenges and reflect individual coping strategies of growing up alongside a chronically ill sibling (quote 4 l).

#### Navigating communication—between the medical and family systems (Table 6)

**Table 6:** Navigating communication—between the medical and family systems

Quote	Quotation		Participant
5a	“If you don’t take action yourself, you get very little support. If you ask, yes—then you get answers. But very little comes on its own.”		Parent 6
5b	“I wished that they inform us when something is unclear. Or that they talk to us, explain the situation, and not always present us with a done deal and then hand over some information.”		Parent 4
5c	“Especially because this topic (the disease) always seems to be swept under the rug. No one really wants to talk about it in concrete terms. (…).”		Sibling 2
5d	“(…) Especially with the thought that another transplant might be needed, I would really appreciate the opportunity to speak with the healthcare team. I’d like to talk about how things went last time and what it might look like if we had to go through it again—It would really help to have that kind of exchange.”		Sibling 2
5e	“I’m well informed—enough to keep my kidney alive.”		Patient 1
5f	“I think sometimes it’s actually good not to know everything, because then you don’t worry so much.”		Patient 6
5 g		“I feel most comfortable when it’s not forced on me, but rather when I know what I want to know, and I can just stay out of everything else.”	Sibling 6
5 h		“Honestly, I know what I need to know, and I’ve never really thought much about whether I should know more.”	Sibling 3
5i		“There wasn’t really any communication among the doctors, so as a parent I basically had to memorize all the knowledge and the entire case file—without coming across as a know-it-all, because they (HCP) didn’t want to be lectured, you know? (…) That’s really when it started—me being pushed into this role of the one who holds everything together.”	Parent 5
5j		“Those things can be really nerve-racking, you’re dealing with your own fears, but at the same time you have to be strong for the child. They have their own questions and fears too.”	Parent 6
5 k		“At the beginning, you’re really in a kind of tunnel, so much information comes at you all at once, you just can’t process it all. And you’re really overwhelmed by all the medical terminology. And in that moment—I mean yes, of course they have to give you the information—but I was overwhelmed. I think I would have preferred if, later on – afterwards—everything had somehow been talked through or processed again.”	Parent 6
5 l		“I think, honestly, I didn’t really want anything at the time, because getting into the emotions during those high-functioning phases would’ve felt counterproductive. There wasn’t much time to process anything. And I have this feeling that, if I ever do have time for myself at some point in life, I might be able to revisit it and process it.”	Parent 5
5 m	“I think it’s good to be shown different perspectives, to be given hope and to see that there are possibilities. But I also think it’s important to communicate honestly and to say: okay, things might not go as planned, and then we need to figure out what we can do. Of course, it’s good to stay hopeful and optimistic and to focus on the positive. But it helps a lot more if you also acknowledge the negative possibilities and include them in the decision-making.”		Sibling 2

Participant numbers correspond to family units (e.g., Patient 1, Sibling 1, and Parent 1 belong to the same family)

Most family members in a caregiving role perceived medical communications insufficient and reactive, with information typically provided only when explicitly requested (quotes 5a–5c). Siblings who took care responsibilities expressed frustration about being excluded from medical discussions and voiced a stronger need for information, inclusion, and participation in decision-making (quote 5 d).

Patients and siblings who were not actively involved in caregiving reported feeling sufficiently informed and generally satisfied with the level of medical information they received. Some expressed relief at not knowing everything, as this helped them worry less and “stay out” of disease related responsibilities (quotes 5e–5 g). They did not actively reflect on their level of knowledge and acknowledged certain gaps without expressing a desire for more information (quote 5 h). These siblings preferred to remain in a distant and “safe observer role” without being confronted with disease-related information (quote 3f).

Caregivers described themselves as the center of information flow—mediating between medical professionals and the rest of the family—while also managing their own and their child’s emotional responses (quotes 5i and 5j).

Parents reported difficulties processing medical information, especially when receiving the diagnosis or during acute crisis. Parents expressed their wish for ongoing communication to support their understanding of complex medical information, as well as help manage the psychological distress which often occurs in these situations (quotes 5 k and 5 l).

Families expressed a desire for transparent and clear structured communication also with mentioning the negative aspects of a medical condition to facilitate decision-making (quote 5 m).

## Discussion

This study demonstrates how pediatric CKD affects the entire family and how it is managed by each individual family member, highlighting the illness’s broader impact on the familiar system and the need for family-sensitive approaches in pediatric nephrology care.

Many families understood CKD as a lifelong condition, yet post-transplant stability was interpreted by most of the families as a sign of recovery or cure, rather than as one step within a chronic and progressive disease course [16]. Similar misconceptions have been described in several pediatric diseases, including oncology and transplantation contexts, where perceived “recovery” can unintentionally reduce future preparedness [17].

Coping strategies varied across families but tended to align within individual family units, reinforcing that pediatric CKD is managed collectively, rather than individually [2, 9, 17]. When parental caregiving roles were disrupted—due to illness, social challenges, or loss—responsibility was redistributed within the family. Siblings stepped into a flexible adaptive role taking on responsibilities that helped stabilize family functioning. This pattern resonates with risk of parentification described in other chronic illness settings [8, 18, 19]. Our findings add a positive way of coping: Siblings framed caregiving as meaningful, fostering emotional maturity and identity development through reciprocity and recognition. When such reciprocity was absent, siblings described emotional strain [20]. This duality highlights the importance for pediatric nephrology teams to acknowledge both the vulnerabilities and potential strengths inherent in siblings’ involvement.

Across roles, siblings reported reduced parental attention and described navigating challenges independently—an experience also mirrored in research on siblings of children with other chronic and life-limiting conditions [8]. Building on our findings and this body of evidence, we suggest that healthcare professionals (HCPs) also focus on the needs of siblings and educate parents on how to better address to siblings’ needs [21].

Communication within the healthcare system emerged as a central challenge, particularly during acute phases of the illness. In line with Agerskov et al., parents described acting as mediators, between their family and the healthcare team—an added responsibility that intensified stress during disease trajectory [22]. Lack of timely or transparent information amplified this burden. These findings align with evidence showing that unclear communication contributes to emotional distress, whereas honest, age-appropriate information fosters psychological safety [23]. HCPs’ hesitancy to proactively address prognosis and the life-limiting nature of CKD aligns with the experiences of caregiving family members who felt insufficiently informed and poorly accompanied in times of crisis [24]. Our data therefore support implementing contingency planning and clearer communication pathways to enhance families’ preparedness and reduce crisis-related overwhelm.

The perceived need for information varied according to the degree of caregiving responsibility rather than the medical condition itself. Patients who were not involved in management tasks felt sufficiently informed, while siblings who assumed responsibilities preferred to stay uninvolved in medical matters. This supports previous work indicating that information preferences depend on caregiving burden, reinforcing the need for individualized approaches [20].

To bridge these gaps, ongoing developmentally appropriate education combined with anticipatory guidance should be included in the routine pediatric nephrology care. Structured pediatric advance care planning might also be a possibility to support the CKD families set realistic expectations and informed, value-based decision-making. Recent studies demonstrate that advance care planning is most effective as a continuous, collaborative process within family-centered care, particularly when co-designed by families and HCPs [17, 25]. This process should be facilitated by a consistent contact person within the primary care team [12].

As observed in this study and supported by literature [26], social stress can intensify caregiving burden and complicate daily family life with CKD. Pediatric palliative care models highlight the importance of providing psychosocial support and early interdisciplinary involvement in families facing complex stressors in such cases [25, 27]. Screening for family-level risk factors could help identify those in need of intensive interdisciplinary care.

While some recurring coping tendencies appeared across families, the study was not designed to classify families or compare them systematically. We therefore refrain from developing a typology and instead highlight this as a direction for future research, consistent with prior work suggesting that family-level coping frameworks may support understanding of communication patterns and care needs in chronic pediatric illness contexts [28].

## Limitations

This study has several limitations. Only German-speaking families were included, limiting cultural diversity. Most participants were stable after several years of being post-transplant, which may influence disease awareness and coping strategies—limiting insights into acute phases such as dialysis or life-threatening complications. Since all family members were interviewed and aware of our research, some hesitated to speak openly about their relationships, fearing they might be unfair to a relative or that disclosed information could be shared, despite assurances of anonymity and written consent. Additionally, some interviews were conducted by phone, meaning on the one hand the interviewer had never met the participant in person, which may have made building trust and rapport more challenging and on the other hand “being alone” could not be verified by the interviewer. A further limitation of this study is the ≥ 14-year age requirement, which limits the applicability of our findings to families with younger children, whose developmental stage could shape different communication patterns and coping processes. Given the limited sample size, potential diagnosis-specific differences may not have been detectable. Last, socioeconomic status was not systematically assessed in this study, which restricts conclusions regarding potential socioeconomic influences on family dynamics.

## Conclusion and future directions

This study underscores the need to treat families—not just individual patients—as units of care in pediatric CKD. Effective care for these families requires clear, ongoing communication, structured advance care planning, and systematic support for siblings and other vulnerable family members.

To translate these findings into practice, pediatric nephrology teams could strengthen family-centered care by implementing concrete measures supported by evidence across chronic illness fields, where structured communication and early psychosocial involvement are linked to improved family well-being [10, 29, 30], such measures as (1) appointing a consistent contact person within the care team, (2) integrating developmentally appropriate education and anticipatory guidance into routine visits, (3) proactively offering whole-family consultations (with the patient’s agreement), (4) clear written information summaries, and (5) routine psychosocial screening.

Future research should investigate the dynamics of families experiencing dialysis or acute complications like graft loss and assessment of socioeconomic context, as financial vulnerability may act as an additional stressor influencing family coping.

## Supplementary Information

Below is the link to the electronic supplementary material.

ESM 1 (DOCX 32.9 KB)

## Data Availability

No datasets were generated or analysed during the current study.

## Abbreviations

CKD: Chronic kidney disease
COREQ: Consolidated criteria for reporting qualitative research
HCP: Healthcare provider
HRQoL: Health-related quality of life
