# Supplementary material for: Pediatric advanced chronic kidney disease as a family challenge: coping and communication in daily life
Source: Eur J Pediatr. 2026 Mar 25;185(4):215. doi: 10.1007/s00431-026-06861-2 (PMC13018009; doi:10.1007/s00431-026-06861-2)
Supplement: Supplementary file 1 — (DOCX 32.9 KB) [file 431_2026_6861_MOESM1_ESM.docx]

**Supplementary Information:**

**Pediatric Advanced Chronic Kidney Disease as a Family Challenge:**

**Coping and Communication in Daily Life**

**For the European Journal of Pediatrics**

**Authors:**

Maria Agnes Jonas^1^, Hendrik, Napierala^4^, Nele Kanzelmeyer^5^, Christina Taylan^5^, Nina Kubiak^2,3^, Julia Thumfart^1^

^1^ Department of Pediatric Gastroenterology, Nephrology and Metabolic Diseases, Charité Universitätsmedizin Berlin, Corporate Member of Freie Universität Berlin and Humboldt Universität zu Berlin, Germany

^2^ Department of Pediatric Respiratory Medicine, Immunology and Critical Care Medicine and Cystic Fibrosis Center, Charité Universitätsmedizin Berlin, Corporate Member of Freie Universität Berlin and Humboldt Universität zu Berlin, Germany

^3^ Clinic for Internal Medicine, Psychosomatics/Psychotherapy, Charité – Universitätsmedizin Berlin, Corporate Member of Freie Universität Berlin and Humboldt Universität zu Berlin, Germany

^4^ Institute of General Practice and Family Medicine, Charité Universitätsmedizin Berlin, Corporate Member of Freie Universität Berlin and Humboldt Universität zu Berlin, Germany

^5^ Department of Pediatric Kidney, Liver, Metabolic and Neurological Diseases, Hannover Medical School, Hannover, Germany

^6^ Department of Pediatric Nephrology, Children´s and Adolescents` Hospital, University Hospital of Cologne, Faculty of Medicine, Cologne, Germany

**Supplementary Material 1:** Overview of main categories and subcategories

| Main categories | Subcategories |
| --- | --- |
| CKD as a chronic life-limiting disease | Medical history  Disease perception Education about disease  Self-perception  Needs/Wishes |
| Coping Mechanism | Emotion oriented   - Acceptance - Optimistic - Living the present moment/ realistic - Avoidant/distraction - Ostrich mentality - Living with a pulled handbrake   Problem oriented   - Responsibilities - Life changes due to CKD - Insight gained through CKD - Dealing with one´s lifetime |
| Family Dynamics | Family approach to dealing with CKD  Relationship within the family (between parents, sibling, patient) Person of trust Need for information |
| Well Sibling | Sibling Relationship  Upbringing  Sibling parentification |
| Quality of life | Daily life School/Studies  Employment/Job  Living situation  Household  Finances Relationships  Hobbies/Interests |
|  |  |

**Supplementary Material 2:** Parents` Interview Guide

**The changes made after the pilot interview are marked in red.

| **Part I: Daily life and challenges** | | | |
| --- | --- | --- | --- |
| Could you start by describing your daily life and the routine you have as a parent? | | | |
| **Check** | **Specific questions** | **Maintenance** | |
| - Family structure - Impact of the illness on family members - Emotional well-being - Responsibilities and obligations | - Since when has your child been ill? - Who is primarily responsible for the daily care – of your sick child, the siblings, and the household? - How has your life changed since your child’s diagnosis – for example, in your relationship, work life, finances, or social circle? - Do you take regular vacations with your children? When was the last time? - What is your current housing situation? | Non-verbal maintenance  Can you describe this in more detail?  What then? | |
| **Part II: Medical Treatment and Palliative Care** | | | |
| What do you understand about your child's diagnosis/illness? | | | |
| **Check** | **Specific questions** | | **Maintenance** |
| - Communicating about the illness | - Who informed you about your child’s diagnosis, and how did you experience that moment? - Do you feel adequately informed and supported regarding your child’s illness and treatment? - What has your experience been with your child’s hospital stays? | | Non-verbal maintenance  How did you feel? |
| **Part III: Coping with the illness** | | | |
| Can you recall a specific situation at home or in the hospital that was particularly challenging for you? Please tell me about it… | | | |
| **Check** | **Specific questions** | | **Maintenance** |
| - Difficulties - Coping strategies - Dealing with/ Feelings towards death and dying | - Do you have any worries concerning your child's illness? If so, why? - Have there been phases during your child’s illness when you thought he or she might die? What was that like for you? - Who or what did you find supportive during your child’s treatment? - Whom do you turn to when facing difficulties? (e.g., professional support, partner, family, friends) - Have you developed any personal strategies that help you in difficult moments? What do they look like? | | Non-verbal maintenance  How did that go ...?  Can you tell me a bit more about this? |
| **Part IV: Needs and Participation** | | | |
| Have you ever thought about your own future or the future of your child? | | | |
| **Check** | **Specific questions** | | **Maintenance** |
| - Personal outlook and attitude - Wishes and hopes - Specific needs - Spirituality, religion, and faith - Fears and concerns | - Career, family planning, fears, wishes – what thoughts do you have about these topics? - What role do faith, religion, or spirituality play in your life? How do they help you cope with everyday challenges? - Have you ever thought about death and dying? In what context did that come up? - What have you learned from your child’s illness? - Have you come across the term “palliative care”? In what situation? What do you understand by it? Why do you think that? - In your opinion, how could palliative care be helpful in your situation? - Where do you see yourself in 10 years? - Has your perspective on time and life changed because of your child’s illness? - What do you wish for in the time ahead? | | Non-verbal maintenance  Can you describe it in more detail?  How did you feel?  Why (not)…? |

**Supplementary Material 3:** Siblings` Interview Guide

**The changes made after the pilot interview are marked in red.

| **Part I: Family structure** | | | |
| --- | --- | --- | --- |
| To begin, could you introduce your family to me? | | | |
| **Check** | **Specific questions** | **Maintenance** | |
| - Position within the family - Development of relationships within the family over time - Satisfaction - Living situation | - Who are you closest to in your family, and why? - How is your relationship with your brother/sister – and has it changed since the diagnosis? - How are caregiving responsibilities divided in your household? (Cleaning, household…)? - How do you feel at home, with your family? - What is your current living situation like? | Non-verbal maintenance  Can you give an example?  Why (not)? | |
| **Part II: Understanding the illness** | | | |
| Could you describe how you experience your sibling’s illness and treatment? | | | |
| **Check** | **Specific questions** | | **Maintenance** |
| - Clarity and openness about the illness in the family - Taboos? - Personal interest in the illness - Satisfaction with medical care | - Has anyone explained your sibling’s illness to you? Who, when, and how? - Do you wish more information about the illness or treatment? - Have you experienced hospitalizations? Did your parents have to leave suddenly? How was that for you? - Have you had any direct contact with doctors or nurses? How was it? | | Non-verbal maintenance  How did you feel?  Anything else? |
| **Part III: Coping with the illness** | | | |
| Can you recall a specific situation at home or in the hospital that was particularly challenging for you? Tell me about it. | | | |
| **Check** | **Specific questions** | | **Maintenance** |
| - Impact of CKD on daily life - Personal challenges - Role of spirituality, religion, or faith | - How does the illness affect your school, free time, friendships, or family life? - Have there been phases during your sibling`s illness when you thought he or she might die? What was that like? - Are you worried about your sibling? Why? - Have you developed any personal strategies to help you in difficult moments? What do they look like? - Whom do you turn to when things get difficult? - Who or what did you find supportive during your sibling’s treatment? | | Non-verbal maintenance  Can you tell me a bit more about this? |
| **Part IV: Future Perspectives and Needs** | | | |
| Have you ever thought about your own future or the future of your sibling? | | | |
| **Check** | **Specific questions** | | **Maintenance** |
| - Coping strategies - Wishes and ideals - Fears and hopes - Outlook on the future | - Career, family planning, fears, wishes – what thoughts do you have about these topics? - What role do faith, religion, or spirituality play in your life? If so, how do they help you cope with everyday challenges? - Have you ever thought about death and dying? In what situation did that come up? - Have you ever heard of "palliative care"? In what context? What do you understand by it? - What have you learned from your sibling’s illness – for yourself? - What have you learned from your sibling’s illness? - Looking ahead – where do you see yourself in 10 years? | | Non-verbal maintenance  Can you describe it in more detail?  How did you feel?  Why (not)…? |

**Supplementary Material 4:** Patients` Interview Guide

**The changes made after the pilot interview are marked in red.

| **Part I: Illness History and Family Context** | | | | |
| --- | --- | --- | --- | --- |
| To start, could you tell me about your illness and what it's like to live with it? | | | | |
| **Check** | **Specific questions** | | **Maintenance** | |
| - Role within the family - Satisfaction with family relationships and its development - Emotional experience related to the illness | - Since when have you known about your illness, and who told you? How was that for you? - Who are you closest to in your family? How are they handling it? - Has your relationship with family members changed since the diagnosis? - Whom do you turn to when things get difficult? - How do you feel at home, with your family? | | Non-verbal maintenance  Can you give an example?  Why (not)? | |
| **Part II: Challenges and Coping with the Illness** | | | | |
| Could you describe a typical day or week in your life? | | | | |
| **Check** | **Specific questions** | | | **Maintenance** |
| - Transparency and openness within the family - Personal interest in one's own illness - Perception of the Illness | - What do you find difficult in your situation? What helps you? - Have you developed personal coping strategies? What do they look like? - How does the illness affect your: Everyday life (school, well-being, relationships, future plans…) | | | Non-verbal maintenance  Can you tell me a bit more about this? |
| **Part III: Quality of Life and Satisfaction with Care** | | | | |
| Would you like to tell me something about yourself before you got ill? | | | | |
| **Check** | **Specific questions** | **Maintenance** | | |
| - Involvement in treatment and care - Openness or taboos in the family - Feelings of empathy, pity, indifference… (self or others) | - Have you experienced moments during your illness when you thought you might die? What was that like for you? How did you feel? - Who or what did you find supportive during those times? - Was there anything during your treatment where you thought, “That went really well – that was especially good”? | Non-verbal maintenance  How did you feel?  Anything else? | | |
| **Part IV: Future Perspectives and Needs** | | | | |
| Have you ever thought about your future? | | | | |
| **Check** | **Specific questions** | **Maintenance** | | |
| - Wishes, hopes and fears - Future perspectives - Spirituality or religion | - Career, family planning, fears, wishes – what thoughts do you have about these topics? - What role do faith, religion, or spirituality play in your life? If relevant: how do they help you cope with everyday challenges? - Have you ever thought about death and dying? In what context or situation did that come up? - Have you heard of “palliative care”? In what context? What do you understand by it? - What have you learned from living with your illness? - Where do you see yourself in 10 years? | Non-verbal maintenance  Can you describe it in more detail?  How did you feel?  Why (not)…? | | |
